# Supplementary material for: MetaRibo-Seq measures translation in microbiomes
Source: Nat Commun. 2020 Jun 29;11:3268. doi: 10.1038/s41467-020-17081-z (PMC7324362; doi:10.1038/s41467-020-17081-z)
Supplement: Supplementary file 10 — Supplementary Data 7 [file 41467_2020_17081_MOESM10_ESM.zip › File2/Confidence_High_Taxonomy/279885_out.krona.html]

Javascript must be enabled to view this page.

members
magnitude
magnitudeUnassigned
count
unassigned
taxon
rank

279885\_out

7

7
2
superkingdom

1239
7
phylum

class
7
186801

order
7
186802

family
3
186803

572511
3
genus


SRS055982\_contig\_number\_contig-100\_333.100900SRS149879\_contig\_number\_18366
2
40520
species

species
1
418240

SRS105153\_contig\_number\_34133

4
family
541000

genus
4
1263

species
2
2293207

SRS017521\_contig\_number\_49078SRS017916\_contig\_number\_14829

species
1
2293198

SRS050422\_contig\_number\_35467

1
species
2293185

SRS104311\_contig\_number\_36955
